# Bœnninghausen’s Aphorisms of Hippocrates

**Published:** 1887-07

**Authors:** 


### BŒNNINGHAUSEN'S APHORISMS OF HIPPOCRATES.

Extracts Translated by Dr. A. McNeil, San Francisco.

### Apoplexy.

We briefly mention a case of the cure of this disease, which hastened the conversion of an aged allopathic physician. During the year 1831 Drs. Feristing and Lutterbeck, the latter of whom was formerly physician-in-ordinary to the celebrated Prince Blücher, spent every Saturday evening with us in conversation on Homœopathy. It was on one of these evenings that our cook, who is still living and well and very old, was stricken with apoplexy. All three of us hastened to her, when the aged allopaths were hastily drawing their lancets to open a vein, which we prevented, and instead gave a small dose of Aconite, which was perfectly indicated in her case. In a few minutes the unconscious patient had regained her senses, but there was now revealed a *paralysis* of the entire *left side of the body*. The half-convinced Dr. L. could not believe our assurance that by refraining from the lancet the paralysis would soon be removed. In the meantime the paralytic was carried to bed, and received an hour afterward a small dose of Cocculus. At seven o'clock the next morning Dr. L. returned to learn the result, and in answer to the doorbell, the door was opened, to his great astonishment, by the same person who had been struck by *apoplexy with paralysis of one side* in her own person, and she assured him that during the night the paralysis had entirely disappeared, and that she felt as well as ever. In consequence Dr. L. was thoroughly converted, and remained a zealous and true homœopath till his death.

